# Reply: Under-Capture of Candidates Supported by Temporary Mechanical Circulatory Support in the OPTN STAR Registry

**DOI:** 10.1016/j.jacadv.2026.103215

**Published:** 2026-09-10

**Authors:** Nicole Cyrille-Superville, Priyesh A. Patel, Snehal Patel, Onyedika Ilonze, Adam D. Devore

**Affiliations:** a Sanger Heart and Vascular Institute, Atrium Health, Charlotte, North Carolina, USA; b Division of Cardiology Northwell Health, Department of Medicine, New York, USA; c Krannert Cardiovascular Research Center, Indiana University School of Medicine; d Department of Medicine, Duke University School of Medicine, Durham, North Carolina, USA

We are grateful for the authors' interest in our study^1^ and their recognition of its importance to the field. We also appreciate their thoughtful letter highlighting an important consideration regarding the temporary mechanical circulatory support (MCS) data included within the Standard Transplant Analysis and Research (STAR) registry from the Organ Procurement and Transplant Network (OPTN).

As noted, device information is obtained from OPTN MCS removal forms and hence may not capture candidates that are actively listed at the time of data extraction, thereby underestimating the absolute prevalence of temporary MCS among waitlisted patients. However, there are several reasons why this limitation is unlikely to significantly impact the main findings of our study. Firstly, we examined temporal trends across the allocation periods and sex-based differences among those listed on temporary MCS and not the exact numeric prevalence of these devices across the waitlist. For this limitation to impact our conclusion, delayed capture would need to differ substantially between the sexes and in a manner large enough to affect the overall trend. Notably, other observed sex-specific patterns remained consistent throughout the 8-year study period and even across allocation periods suggesting stable and consistent trends that are unlikely to be affected by minor numeric differences in the STAR data. For instance, women consistently accounted for approximately a quarter of those listed on temporary MCS corresponding to a stable female-to-male ratio of roughly 1:4 over the study period.

Secondly, given the shorter duration of support and waitlist time for candidates bridged with temporary devices, the degree of undercounting described by Ahn et al,^2^ is relatively modest and substantially smaller than the degree of misclassification observed in the durable MCS population. Utilizing similar criteria, the authors reported capturing 2,533 candidates supported on temporary MCS between December 1, 2023, and November 30, 2024, using a December 31, 2024, extraction vs 2,611 candidates with data extraction performed December 31, 2025. In accordance with OPTN policy, a patient who receives a deceased donor heart transplant must be removed from the waitlist within 24 hours of the transplant;^3^ hence, although this increase of 78 candidates demonstrates a limitation of the STAR MCS file, the reported difference is small in magnitude (3%) and is unlikely to impact overall trends unless significant sex-specific differences existed in the timing of waitlist removal.

We acknowledge that studies utilizing the STAR data should highlight the source of MCS ascertainment, date of data extraction relative to cohort closure, and incorporate available status justification forms to ensure transparency and provide a more complete characterization of MCS utilization. Although this provides context for our findings, our study’s overall conclusion that significant sex-based differences exist among patients bridged with temporary MCS to orthotopic heart transplant is not materially altered. Future analyses within multiple data sets may help confirm the consistency of findings.
